# Recurrent synovial chondromatosis in the hand leading to second ray amputation: A case report

**DOI:** 10.1016/j.ijscr.2025.111164

**Published:** 2025-03-17

**Authors:** Mallory Rongstad, Jarod Moyer, Rachel Mifflin, Kevin Condit, Kurt Rongstad

**Affiliations:** a Drexel University College of Medicine, United States; b University of Wisconsin Hospitals and Clinics, United States

**Keywords:** Synovial chondromatosis, Ray amputation, Metacarpophalangeal joint, Cartilaginous nodules, Case report

## Abstract

**Introduction:**

Synovial chondromatosis is a rare, benign metaplasia of the synovium characterized by the formation of cartilaginous nodules. This condition can lead to joint stiffness, pain, and damage. It is most common in the knee but has been described in various joints, including the hand. Due to its low prevalence and nonspecific symptoms, delayed diagnosis may lead to complications such as local invasion, pain, osteoarthritis, and, rarely, malignant transformation. Surgical intervention is often indicated after conservative management fails.

**Case report:**

This report describes the complex case of synovial chondromatosis of the second metacarpophalangeal (MCP) joint in an 18-year-old female. Multiple surgical interventions culminated in amputation of her second ray for definitive management.

**Discussion:**

This case underscores the complexities of diagnosing and managing synovial chondromatosis, particularly in rare locations like the hand. Despite radiographic and pathological evidence, a definitive diagnosis was delayed, contributing to prolonged morbidity. The aggressive and recurring nature of this case highlights the need to consider synovial chondromatosis in refractory hand and finger joint pain, regardless of patient age or gender. Questions remain about whether earlier aggressive intervention could reduce recurrence or if earlier amputation could improve outcomes.

**Conclusion:**

This case highlights the challenges of diagnosing and managing recurrent synovial chondromatosis in the hand. Early recognition and aggressive intervention may prevent prolonged morbidity and functional impairment. Further research is needed to determine whether earlier amputation could improve outcomes in refractory cases.

## Highlights

• Synovial chondromatosis of the hand is uncommon and challenging to diagnose.
• There is risk of recurrence and functional impairment even after multiple surgeries.
• Earlier aggressive surgery or amputation could improve patient outcomes.

## 1 Introduction

Synovial chondromatosis is a rare, benign metaplasia of the synovial joint lining characterized by the formation of cartilaginous nodules in the synovial sheath. These cartilaginous nodules can become pedunculated, break loose, and potentially ossify leading to joint damage [1,2]. The loose bodies can vary in size and number [3]. Synovial chondromatosis is most common in the knee, and is uncommon in the hand [1,3,4]. The condition predominantly affects men aged 30–60 years [5].

Diagnosis involves physical exam, radiologic imaging, and pathological investigation. Radiographs are frequently pathognomonic for synovial chondromatosis, showing homogenous intraarticular calcifications distributed throughout the joint [6].

Because of its low prevalence and nonspecific symptoms, diagnosis can be delayed and may stall treatment [1]. Untreated synovial chondromatosis can result in significant complications, including invasion of nearby tissues, severe pain, osteoarthritis, and in rare cases, malignant transformation into secondary synovial chondrosarcoma [7,8]. Therefore, after a trial of conservative management, surgical interventions are often indicated, such as arthroscopic removal of the loose bodies, open synovectomy, or in severe cases, amputation. Synovectomy with free body removal is often preferred to minimize recurrence [9].

The following report, which is in line with the SCARE criteria, describes a complex and progressive case of synovial chondromatosis of the second metacarpophalangeal (MCP) joint in a young female patient [10,11]. This is a rare demographic and location for this pathology, as outlined above. The patient required multiple surgical interventions which culminated in an amputation of her second ray for pain relief and functional improvement.

## 2 Case description

The patient, a healthy 18-year-old, right-hand-dominant female, presented with a four-month history of pain and swelling in the left second MCP joint. She denied any antecedent trauma or systemic symptoms. Radiographs demonstrated identified a small, bony ossicle at the volar aspect of the left second MCP joint. She received a corticosteroid injection which provided minimal relief. Pain persisted over the following three years and MRI was obtained which revealed edema around the metacarpal head, significant synovitis, and loose bodies which were thought to represent sesamoids. Given these findings, the patient underwent surgical excision of the loose bodies with synovectomy. Histopathologic examination showed benign cartilage, bone, and inflamed synovium. This procedure provided minimal clinical improvement.

**Fig. 1:**
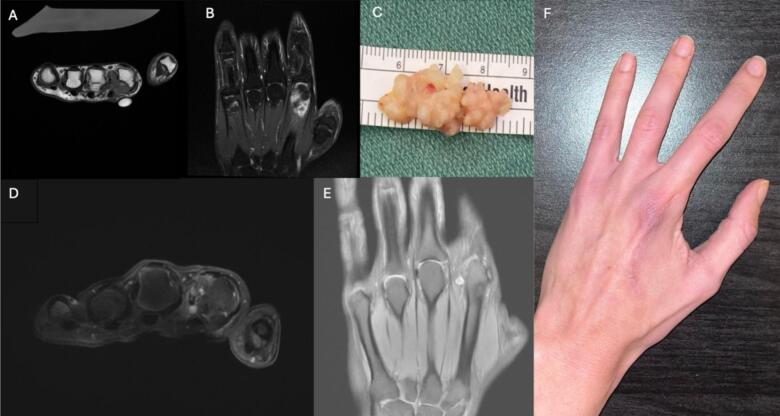
A. MRI images showing hyperintensity and heterogeneous enhancement palmar to the second metacarpal head and neck, T1 without Contrast B. Short Tau Inversion Recovery (STIR) MRI C. Gross specimen of multilobulated mass measuring 0.6 × 0.5 × 3.0 cm D. MRI demonstrating volar synovitis and chondromas, T2 fat saturation without contrast E. T1 fat saturation with contrast F. Patient's hand two years post ray amputation.

Two years later, repeat MRI was obtained and demonstrated a 1.8 × 1.7 × 1.6 cm irregular focus of heterogeneous enhancement communicating with the second MCP joint, consistent with synovitis (Fig. 1A, B). There were no new erosions identified. Surgical intervention was pursued, with an extended palmar approach to the second MCP joint with a complete excision of a 0.6 × 0.5 × 3.0 cm firm, rubbery, multilobulated synovial mass (Fig. 1C) and pathology now confirmed the diagnosis of synovial chondromatosis. Following the surgery, the patient experienced nearly full resolution of symptoms with both improved range of motion and reduced pain. Approximately a year post-synovectomy, the patient developed new dorsal second MCP joint pain that progressed over 2 years, leading to a third surgery that removed two 4–5 mm loose chondromatous bodies. This procedure resulted in further limitation in range of motion, but brought about a significant improvement in pain.

Over the next five years, the patient described progressive pain and deteriorating range of motion, significantly impacting daily life. Another MRI was obtained which showed recurrent volar synovitis and chondromas in the periarticular soft tissues (Fig. 1D, E). There was also edema within the second metacarpal head with erosions on the ulnar aspect.

Due to chronic disease progression and limited success with previous treatments, the patient and her care team discussed alternative treatment options. Prior to considering ray amputation, the patient had already undergone multiple synovectomies and surgeries aimed at removing loose bodies and addressing synovitis. However, given the aggressive, recurring nature of the disease and the limited functional improvement from prior surgeries, repeat synovectomy was no longer considered a viable option. Therefore, ray amputation was performed. Intraoperative findings revealed that the synovial chondromatosis was fully contained within the resected second ray, with no neoplastic changes identified in pathology.

At one-year follow-up, the patient reported complete resolution of pain and significantly improved function, with no limitations in daily activities (Fig. 1F). Prior to amputation, she had anticipated improved function due to the chronic pain she had experienced. Following the amputation, the patient's symptoms were fully resolved, demonstrating the efficacy of the procedure in alleviating pain and restoring function.

## 3 Discussion

This case underscores the complexities involved in diagnosing and managing synovial chondromatosis, especially when occurring in rare locations such as the hand. Despite having radiographic and pathological evidence consistent with synovial chondromatosis following her initial surgery, the patient did not receive a definitive diagnosis, or experience significant relief, until her second surgery, which involved a complete synovectomy [6]. The progressive nature of the disease, along with the risk of recurrence and potential for neoplastic transformation, highlights the need to include synovial chondromatosis in the differential diagnosis for refractory hand and finger joint pain, irrespective of the patient's age or gender [7].

Furthermore, the aggressive and chronically recurring nature of this case is notable. A 2021 article conducted a literature review and found 23 publications from 2008 to 2019 that discussed synovial chondromatosis of the hand or finger [4]. Of these 23 publications, there were four cases of documented recurrence. Another report found that of 16 cases of tenosynovial chondromatosis, 14 patients experienced one or more recurrence after surgical procedures [12]. The risk of recurrence is a significant challenge, with rates varying between 3 and 60 % in the literature. While recurrence remains common after the current first-line surgical interventions with synovectomy and the removal of loose bodies, there is no specific mention of early amputation in the literature as a common or first-line treatment. However, this case presents an opportunity to explore the potential role of amputation when the disease becomes chronic and resistant to repeated surgeries.

The rarity of this condition contributed to a 5-year delay in diagnosis for our patient, who then experienced recurrent pain and functional impairment for 13 years before ultimately resulting in amputation. This delayed diagnosis and prolonged disease course align with the literature, where recurrence is common despite repeated surgeries. However, most cases involve less radical treatment and continued management with joint-preserving approaches. This case prompts two critical questions regarding the management of recurrent synovial chondromatosis:

1. Could earlier diagnosis and a more aggressive initial resection reduce the risk of recurrence?
2. When should amputation be considered since the disease is progressive and resistant to repeated debridements?

While amputation is not commonly performed early in the treatment of synovial chondromatosis, our case highlights its potential role when the disease is chronic, progressive, and resistant to multiple surgeries. Amputation might improve patient outcomes by alleviating symptoms and reducing the need for recurrent surgical interventions. Although ray amputation leads to permanent loss of the affected digit, in our case, it resulted in substantial functional recovery and pain relief. This suggests that index ray amputation of the non-dominant hand could be a valuable strategy in cases with significant disease burden and poor prognosis from repeated surgeries. However, ray amputation requires careful consideration of both functional and psychological impacts. Further research is needed to assess the effectiveness of early aggressive intervention and to compare the outcomes of early versus delayed amputation in cases of recurrent synovial chondromatosis.

## 4 Conclusion

This case highlights the challenges of diagnosing and managing recurrent synovial chondromatosis in the hand. Early recognition and aggressive intervention may prevent prolonged morbidity and functional impairment. Further research is needed to determine whether earlier amputation could improve outcomes in refractory cases.

## Registration of research studies

N/A.

## Consent

Written informed consent was obtained from the patient for publication of this case report and accompanying images. A copy of the written consent is available for review by the Editor-in-Chief of this journal on request.

## Ethical approval

This study is exempt from ethical approval from the University of Wisconsin Department of Orthopedics and IRB: Case reports of 3 or fewer individuals generally do not meet the regulatory definition of research because they would not qualify as a systematic investigation that contributes to generalizable knowledge. Medical case reports are done to highlight a unique treatment, a unique case, or a unique outcome. Case reports are generally done by retrospective review of the medical record. Nothing is done to the patient for a “research” purpose. Statistics are not used and the case report generally only describes existing clinical data or procedures. IRB review of such case reports is therefore not required.

## Guarantor

Mallory Rongstad.

## Funding

We received no funding.

## Author contribution

Mallory Rongstad – writing the paper, data collection.

Jarod Moyer – writing the paper.

Rachel Mifflin – data collection.

Kevin Condit – editing the paper.

Kurt Rongstad – PI, data collection, overseeing project.

## Declaration of competing interest

We have no conflicts of interest to disclose.
